# Nerpa: A Tool for Discovering Biosynthetic Gene Clusters of Bacterial Nonribosomal Peptides

**DOI:** 10.3390/metabo11100693

**Published:** 2021-10-11

**Authors:** Olga Kunyavskaya, Azat M. Tagirdzhanov, Andrés Mauricio Caraballo-Rodríguez, Louis-Félix Nothias, Pieter C. Dorrestein, Anton Korobeynikov, Hosein Mohimani, Alexey Gurevich

**Affiliations:** 1 Center for Algorithmic Biotechnology, Saint Petersburg State University, Saint Petersburg 199004, Russia; o.kunyavskaya@spbu.ru (O.K.); a.tagirdzhanov@spbu.ru (A.M.T.); a.korobeynikov@spbu.ru (A.K.); 2 Scientific Center for Information Technologies and Artificial Intelligence, Sirius University of Science and Technology, Sochi 354340, Russia; 3 Department of Higher Mathematics, Saint Petersburg Electrotechnical University “LETI”, Saint Petersburg 197376, Russia; 4 Collaborative Mass Spectrometry Innovation Center, Skaggs School of Pharmacy and Pharmaceutical Sciences, University of California San Diego, La Jolla, CA 92093, USA; acaraballorodriguez@ucsd.edu (A.M.C.-R.); Louis-Felix.Nothias@unige.ch (L.-F.N.); pdorrestein@ucsd.edu (P.C.D.); 5 Department of Mathematics and Mechanics, Saint Petersburg State University, Saint Petersburg 199004, Russia; 6 Computational Biology Department, School of Computer Sciences, Carnegie Mellon University, Pittsburgh, PA 15213, USA

**Keywords:** natural products, nonribosomal peptides, genome mining, biosynthetic gene clusters, bioinformatics, mass spectrometry, software, machine learning

## Abstract

Microbial natural products are a major source of bioactive compounds for drug discovery. Among these molecules, nonribosomal peptides (NRPs) represent a diverse class of natural products that include antibiotics, immunosuppressants, and anticancer agents. Recent breakthroughs in natural product discovery have revealed the chemical structure of several thousand NRPs. However, biosynthetic gene clusters (BGCs) encoding them are known only for a few hundred compounds. Here, we developed Nerpa, a computational method for the high-throughput discovery of novel BGCs responsible for producing known NRPs. After searching 13,399 representative bacterial genomes from the RefSeq repository against 8368 known NRPs, Nerpa linked 117 BGCs to their products. We further experimentally validated the predicted BGC of ngercheumicin from *Photobacterium galatheae* via mass spectrometry. Nerpa supports searching new genomes against thousands of known NRP structures, and novel molecular structures against tens of thousands of bacterial genomes. The availability of these tools can enhance our understanding of NRP synthesis and the function of their biosynthetic enzymes.

## 1. Introduction

Nonribosomal peptides (NRPs) are promising natural sources of antibiotics, immunosuppressants, anticancer agents, toxins, siderophores, pigments, and cytostatics [1]. Starting from penicillin [2], researchers revealed the chemical structure of several thousand NRPs [3]. However, the mechanism of their biosynthesis remained unclear until the end of the 20th century [4,5]. Currently, only 10% of known NRPs are associated with genes encoding them [6].

In contrast to regular ribosomal peptides encoded by short genes (about 1000 nucleotides long for prokaryotes), NRP production involves the coordinated action of giant biosynthetic gene clusters (BGCs) spanning hundreds of thousands of nucleotides. These BGCs encode multi-modular proteins, NRP synthetases (NRPSs), responsible for the assembly of NRP products. An NRPS BGC consists of one or more genes composed of NRPS modules wherein each module incorporates an amino acid into a final product [7]. Each module usually contains an Adenylation (A) domain responsible for recognizing and activating the specific amino acid. Modules also require Peptide Carrier Protein (PCP) and Condensation (C) domains that load and elongate the NRP scaffold. Modules may include Epimerization (E), Methylation (M), and other domains responsible for modifications of the incorporated amino acid. The first (initiation) module of an NRPS BGC may start with a specialized C Starter (CS) domain acylating the first amino acid with a fatty acid [8]. The last (termination) module often ends with a thioesterase (TE) domain releasing the NRP product.

The cracking of nonribosomal code [5] enabled the appearance of software for predicting NRP products from microbial genomes, such as NP.searcher [9], PRISM [10,11] and the currently state-of-the-art and actively maintained antiSMASH pipeline [12,13,14,15,16]. The core element of these genome mining pipelines is substrate specificity predictors trained on a set of A domains with known specificities, for example, NRPSpredictor2 [17] and SANDPUMA [18]. These algorithms report a list of substrates possibly recruited by the A domains and scores reflecting the confidence that the particular amino acid was selected. However, many of the NRP substrates are under-represented in the training data of annotated domains used by these algorithms. As a result, the tools may generate low score (unreliable) specificity predictions if an observed A domain sequence differs from all A domains in the training data. Moreover, even if all the A domain specificities are correctly identified in a BGC, it might be difficult to predict the final compound due to ambiguities in the order of domains in the assembly line.

In non-collinear NRPS assembly lines, the order of genes in a BGC may deviate from their activation order due to rearrangement, skipping, stuttering, and iterative reuse of genes [19,20]. Besides that, a linear peptide sequence produced by an NRPS assembly line often undergoes macrocyclization or other tailoring reactions, resulting in a cyclic, branch-cyclic, or even more complex structure [21]. Thus, predicting the correct NRP structures solely from genomics data remains a challenge despite progress in genome mining [11,16,22]. As a result, genome-based predictions require validation through orthogonal data, such as mass spectra [23,24,25] or chemical structure databases [6,26].

In a recent landmark study, MIBiG, a community-driven database of BGCs and their experimentally validated molecular products has been collected [27]. As of August 2021, MIBiG has 605 NRP-related BGCs [28]. At the same time, the database of antiSMASH-predicted gene clusters contains almost 12,000 NRPS BGCs fragments, predominantly without links to known compounds [29]. Moreover, this number is rapidly growing with advances in sequencing technologies and genome mining software. Linking these automatically generated predictions to databases of previously isolated NRPs, such as Norine [30], is a challenging computational problem. In particular, antiSMASH only allows slow semi-manual queries of roughly predicted NRP scaffolds to the Norine search engine. The feature is restricted to the single database and lacks the support for non-collinear NRPS assembly lines.

The SeMPI web server addressed some of the antiSMASH limitations [26]. It provides a genome mining pipeline focusing on high-quality scaffold predictions for NRPs and type I polyketides. The authors demonstrate their tool outperforms antiSMASH v5 in cluster detection and domain substrate prediction accuracy. The SeMPI pipeline can screen the predicted scaffolds in public natural product databases, such as MIBiG, Norine, and StreptomeDB [31]. However, the tool is available only as a web server, and it is not applicable to high-throughput searches.

GARLIC is a three-step approach to linking known NRPs and polyketides to their BGCs [6]. First, PRISM v2 [10], detects BGCs and predicts the scaffolds. Next, the known chemical structures are reduced to linear sequences of residues using the GRAPE retro-biosynthetic engine [6]. Finally, GARLIC matches the two sets of retrieved sequences against each other using global alignment [32]. In contrast to the previous methods, GARLIC is available as a command-line tool and thus applicable to arbitrary databases and genomes. It also accounts for non-collinear NRPS assembly lines by trying various gene permutations within multi-gene BGCs. However, iterative NRPs and other non-collinearity features are ignored. Moreover, GARLIC is prohibitively slow for large BGCs.

Here we present Nerpa, a method for screening genomes against NRP databases and linking predicted BGCs to their products. The tool works with non-collinear NRPS assembly lines and outperforms GARLIC in accuracy and efficiency. We demonstrate Nerpa performance by searching 13,399 bacterial genomes against 8368 NRPs. The run revealed known and novel BGC-NRP pairs, including a putative ngercheumicin BGC, experimentally validated via mass spectrometry. Nerpa is freely available as a command-line tool from http://cab.spbu.ru/software/nerpa (accessed on 30 September 2021).

## 2. Results

### 2.1. Outline of the Nerpa Pipeline

**Figure 1:**
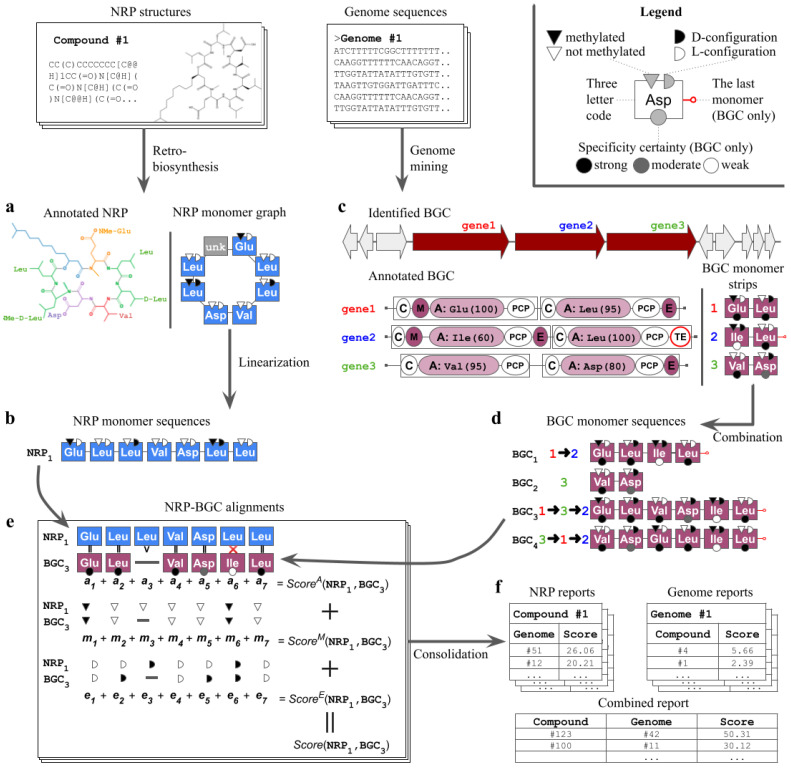
Nerpa pipeline. Structures of nonribosomal peptides (NRPs) are annotated into NRP building blocks (monomers) via retro-biosynthesis (**a**). Nerpa represents the annotated NRPs as monomer graphs. Graph nodes include core amino acids (shown with the three letter code), their stereochemistry (black/white semicircles) and methylation state (black/white triangles). A node may also designate an unknown monomer, such as a lipid tail (grey; labeled ‘unk’). Then, Nerpa linearizes graphs into sequences of monomers (**b**). Parallel to this, genome mining software processes input genome sequences to identify tentative biosynthetic gene clusters (BGCs) of NRP synthetases (NRPSs) (**c**). The software annotates core NRPS genes into modules and domains. Adenylation (A) domains are responsible for selection of the amino acids, while methylation (M) and epimerization (E) domains are responsible for modifications. The thioesterase (TE) domain is the last domain in the NRPS assembly line. For each A domain, predicted amino acids and their scores are also shown. Nerpa stores NRPS gene annotations as short strings of monomers. Here, black, grey, and white circles stand for specificity predictions with high, mediocre and low scores. These strings are further combined into complete monomer sequences reflecting the putative assembly lines of the BGC (**d**). Next, all NRP and BGC monomer sequences are aligned against each other and scored (**e**). The scoring consists of three independent components reflecting the alignment of core amino acids, their methylations and stereochemistry. Nerpa selects the best scoring combination of sequences as a representative alignment for the given BGC-NRP pair. Top scoring pairs between a single NRP and all genomes, a single BGC and all NRPs, or all genomes against all NRPs are reported (**f**).

Nerpa takes as input an NRP database and nucleotide sequences including complete genomes and draft assemblies (Figure 1). The pipeline starts by detecting (i) linear representations of the database structures (Figure 1a,b), and (ii) tentative NRPS assembly lines along with respective sequences of genome-predicted residues (Figure 1c,d). Afterward, Nerpa (iii) aligns the retrieved sequences against each other in an all-vs-all manner (Figure 1e), and (iv) reports best matches per genome or per NRP (Figure 1f). All steps are described in detail in the Materials and Methods Section. In (i) and (ii), Nerpa relies on the leading third-party NRP retro-biosynthesis and genome mining software, namely rBAN [33] and antiSMASH v5 [16], integrated with the pipeline.

Steps (i)–(iii) operate with NRP building blocks (*monomers*), which include amino acids, lipid tails, and other types of residues. We distinguish between the monomers that originated from the decomposition of NRP structures (*NRP monomers*), and those predicted by genome mining (*BGC monomers*). Each monomer is typically identified by the core amino acid, its stereochemistry (D-/L-configuration), and whether it is methylated or not. BGC monomers additionally include specificity prediction scores. The alphabet of supported core amino acids is the same for both NRP and BGC monomers, and contains 58 residues (Supplementary Table S1).

### 2.2. Database of Putative NRPs

We constructed a database of putative NRPs, further referred to as pNRPdb, by combining all compounds from PNPdatabase [34], peptidic compounds from the NP Atlas [35], entries with SMILES from Norine [30], and NRP-related structures from MIBiG [28]. Duplicate compounds were removed based on their InChiKey values. Since the PNPdatabase and NP Atlas metadata lacks the classification into NRPs and non-NRPs, some compounds represent other classes of peptidic natural products such as ribosomally synthesized and post-translationally modified peptides (RiPPs). We partially addressed this problem by excluding from pNRPdb all compounds identical to known RiPPs from MIBiG and RiPPDB [36] along with their stereoisomers. We also constrained the database to compounds that include at least two NRP monomers as identified by rBAN.

The resulting database contains 8368 compounds (Supplementary Figure S1). Most of them are putative NRPs and only 1261 are reliably classified as an NRP or NRP-PK hybrid. Source databases metadata allowed us to link most of the compounds to their original producers (Supplementary Figure S2). Nerpa successfully identified the NRP monomer sequence of 7677 (92%) pNRPdb compounds producing 6961 unique sequences. The processing of the remaining 691 compounds failed due to ambiguous monomer graphs resulting from errors in the monomer recognition or complex post-assembly line modifications.

### 2.3. Benchmarking Nerpa Accuracy

A dataset of annotated NRPS BGCs was used to benchmark Nerpa against GARLIC. To exclude hybrid clusters, we constructed the dataset by selecting all MIBiG entries marked as producing NRPs and lacking other annotations. BGCs from nonbacterial sources were also excluded. The final dataset, further referred to as MIBiG_NRP_, contains 194 BGCs. Some of the 194 corresponding NRPs do not contain two rBAN monomers, and therefore are filtered out in pNRPdb. To make the subsequent benchmarking experiment fair, we extended pNRPdb with the 81 missing compounds and refer to the resulting database with 8368+81=8449 structures as pNRPdb+.

Nerpa and GARLIC were used to search MIBiG_NRP_ against pNRPdb+ in an all-vs-all manner. The running commands and software versions for this and following experiments are in Appendix A. A BGC was considered as *correctly identified* by the tool if the corresponding ground truth NRP is among the ten best-scoring hits per the BGC. Such a relaxed definition is needed to account for the presence of close structural variants of a correct NRP in pNRPdb+. These similar structures may belong to the same NRP family and therefore be produced by the same or analogues BGC. As a result, some of the NRP variants may match a BGC with scores better than the score $S_{GT}$ of the ground truth NRP making its rank $r_{GT}$ among the BGC hits greater than 1. Still, $S_{GT}$ should be close to the score $S_{best}$ of the best hit, that is, their ratio $S_{GT}/S_{best}$ should be close to 1.0. In the benchmarking experiment, mean $S_{GT}/S_{best}$ for all correctly identified BGCs is 0.85 (SD = 0.20) for Nerpa and 0.90 (SD = 0.13) for GARLIC; mean $r_{GT}$ is 3.7 (SD = 2.8) for Nerpa and 3.0 (SD = 2.5) for GARLIC.

For each correctly identified BGC, we picked the ground truth identification along with its score as the BGC representative. For the remaining BGCs, the best hit was selected. We further sorted all selected BGC-NRP pairs by score in descending order, separately for Nerpa and GARLIC. For each prefix *i* of the sorted list, we count the number of correctly identified BGCs $Num_{cor}\left[i\right]$ and compute the tool’s false discovery rate (FDR) as

$$FDR\left[i\right]=\frac{i-Num_{cor}\left[i\right]}{i}$$

**Figure 2:**
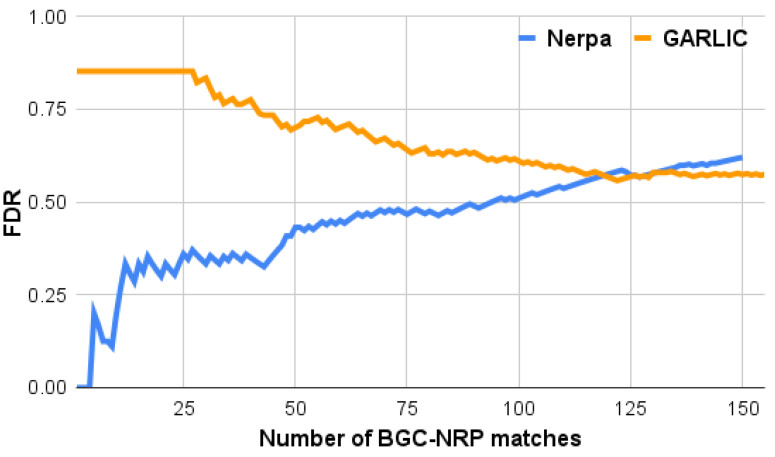
Nerpa (blue) and GARLIC (orange) accuracy in the MIBiG_NRP_ (194 biosynthetic gene clusters, BGCs) against pNRPdb+ (8449 nonribosomal peptides, NRPs) experiment. The *y*-axis shows the tool false discovery rate (FDR) for *x* top-scoring BGC-NRP matches. Matches with tied scores were assigned the common FDR value computed for all these matches together.

Figure 2 shows FDR curves for Nerpa and GARLIC. Nerpa identified 46 correct BGC-NRP pairs with an FDR below 50%. This FDR level corresponds to the Nerpa score of 6.0, which we further selected as the default minimum score threshold. Overall, our tool correctly linked 57 BGCs to their NRPs with a score above 0, which is compatible with the GARLIC result (66). The GARLIC FDR consistently exceeds 50%, meaning our tool better prioritizes correct identifications. For the top 40 matches, Nerpa demonstrates a two times lower FDR than GARLIC (35% versus 78%).

We examined all 23 GARLIC’s false positive matches with the highest possible score (1.0) and revealed its vulnerability towards short BGCs with uncertain A domain specificities (Supplementary Materials). Such uncertain predictions may result from, for example, uncommon substrates (rare amino acids), promiscuous A domains, and shortcomings of the substrate prediction software. Nerpa accounts for scores of the substrate specificity predictions and uses complementary information—substrate’s stereochemistry and methylation state—to successfully deal with BGCs containing uncertain predictions (Section 4).

### 2.4. Benchmarking Nerpa Performance

We downloaded all 13,399 reference and representative bacterial genomes from the NCBI RefSeq database [37] (Supplementary Figure S3). We further sampled five random sets of 100 genomes and matched them against pNRPdb. On average, Nerpa processed the samples 15 times faster than GARLIC and required three times less memory (Table 1). Our tool screened the full set of genomes in less than three days and 20 GB RAM. Using the same computational facilities, processing these data with GARLIC would take approximately three months (Table 1).

### 2.5. Linking Known and Novel BGCs to Their Products with Nerpa

Nerpa identified numerous tentative connections between the RefSeq genomes and pNRPdb structures. We limited our analysis to the BGC-NRP pairs where the BGC genome is the best hit for the particular NRP structure and the NRP structure is also the best hit for the BGC. To be conservative, we kept only the pairs where the genome matches the genus of the original producer of the compound retrieved from the pNRPdb metadata. The resulting set links 117 BGCs to their putative products (Supplementary File S1). For manual validation, we selected pairs with the NRP origin known to the genus level only. The absence of the species level annotation either means a deficiency in the pNRPdb metadata or the current lack of knowledge.

Table 2 represents four BGC-NRP pairs that passed the filtration. Three of the underlying compounds are NRPs and microcystin-LR is an NRP-polyketide hybrid. MIBiG contains one of the identified clusters, the microcystin-LR BGC in *Microcystis aeruginosa*, albeit without a link to the particular compound variant reported by Nerpa (<L-MeSer7>microcystin-LR). NCBI BLAST [38] matched two other cluster sequences to ohmA and sphA (coverage > 95%, identity > 90%), core NRPS genes of recently discovered ohmyungsamycin and stephensiolide BGCs [39,40]. We manually confirmed the Nerpa alignments of the corresponding BGC-NRP pairs are fully inline with the proposed biosynthetic pathways of ohmyungsamycin and stephensiolide. Note that a single run of Nerpa instantly achieved the same goal as the time-consuming semi-manual discoveries of the respective BGCs. The remaining cluster for a putative ngercheumicin BGC in *Photobacterium galathea* is novel since no analogs can be found in any database.

### 2.6. Experimental Validation of Putative Ngercheumicin BGC

We cultured *P. galathea* S2753 [44] in different cultivation conditions and analyzed their extracts by mass spectrometry (experimental details are in Appendix B). The tandem mass spectra (MS/MS) were uploaded to the GNPS platform [45] as part of the MSV000086428 dataset and inspected with the Dereplicator [46], Dereplicator+ [47], and Molecular Networking [45] workflows.

**Figure 3:**
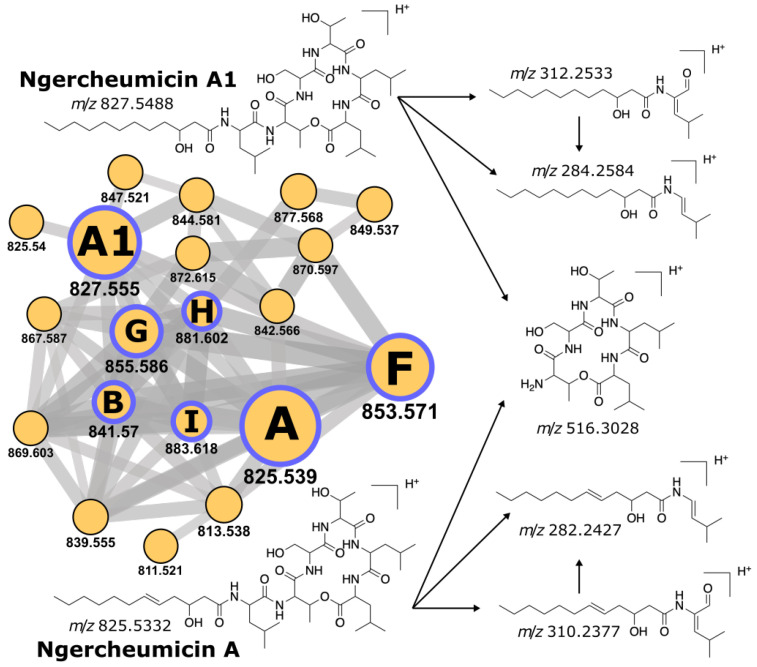
Molecular network for ngercheumicins observed in *P. galathea* extracts (MSV000086428) and the key fragment ions produced for ngercheumicin A and A1. Nodes represent spectral clusters, node size corresponds to the number of clustered spectra, the *m/z* value is specified under the node. Edges connect clusters with a cosine similarity score above 0.7. Purple borders highlight annotated nodes, letters inside the nodes designate particular ngercheumicin variants.

The ngercheumicin family comprises structurally similar variants A-B and F-I produced by *Photobacteria* spp. [43]. Dereplicator annotated numerous *P. galathea* mass spectra as ngercheumicins A and B and their derivatives with *p* values down to $1.1\times 10^{-26}$. Manual validation of the selected spectra confirmed peaks corresponding to the characteristic fragmentation (Figure 3). Dereplicator+ found all six known ngercheumicin variants in MSV000086428 with scores above 30. We deposited their annotated spectra to the GNPS library (accession IDs: CCMSLIB00006710023; 25–28; 33). The ngercheumicin molecular network in MSV000086428 contains all these spectra (Figure 3). The network also contains 13 unannotated nodes that likely represent novel ngercheumicin variants yet to be discovered.

Our experiment validates the production of ngercheumicins by *P. galathea* while the Nerpa alignment suggests its tentative biosynthetic pathway (Supplementary Figure S4). Still, the ultimate confirmation of the putative BGC correctness requires more complex gene knockout or heterologous expression studies.

## 3. Discussion

Breakthroughs in sequencing technologies enabled genome sequencing of thousands of NRP-producing organisms. Although the development of genome mining software, such as antiSMASH, facilitated high-throughput search for BGCs in these data, genes responsible for synthesis of most of the known NRPs still remain undiscovered. Currently, MIBiG, the largest community-curated BGC database, represents a minuscule fraction of all potentially known BGC-NRP pairs. While the expansion of MIBiG is of utmost importance for natural product research, it requires a lot of time-consuming manual work. Here, we demonstrate how a single push-of-a-button Nerpa run can be used for populating the MIBiG repository. First, our tool automatically finds known NRPS BGCs currently missing in MIBiG, such as ohmyungsamycin and stephensiolide. Second, Nerpa reveals novel BGC-NRP connections, which can further undergo experimental validation as shown for ngercheumicin.

The small size of the available training data limits Nerpa accuracy. At the same time, our tool could be used for an iterative extension of the training set via collecting new trustable BGC-NRP pairs in a semi-automated fashion. This will allow both retraining of the current Nerpa parameters and development of a more sophisticated scoring. For instance, we may consider additional monomer modifications such as formylation. Furthermore, the enhanced training data will help to improve Nerpa’s capability to analyse NRP-polyketide hybrids and even polyketides. Still, besides being primarily designed for NRPS BGCs, the current tool correctly linked microcystin-LR, an NRP-polyketide hybrid, to its gene cluster.

We envision two main Nerpa applications in routine NRP research. Nerpa may match recently sequenced bacterial genomes against NRP databases to differentiate BGCs producing known versus novel compounds and thus prioritizing strains for the follow-up studies. Our tool may also screen recently elucidated NRP structures against large genomic databases to find their putative producers NRPS BGCs. The revealed BGCs could be useful for enhancing the NRP synthesis via heterologous expression and for searching or bioengineering novel variants of the compound. To make the tool application fast and convenient, we provide all Nerpa-preprocessed chemical and genomics datasets used in this study. For further convenience, we are preparing a Nerpa web interface that will be integrated with the antiSMASH web services. We believe our software will benefit the community and facilitate the search and discovery of novel bacterial NRPs. Further development of genome mining software will help to extend the Nerpa functionality to other NRP producers such as fungi, plants, and sponges.

## 4. Materials and Methods

### 4.1. Processing of NRP Structures

**Figure 4:**
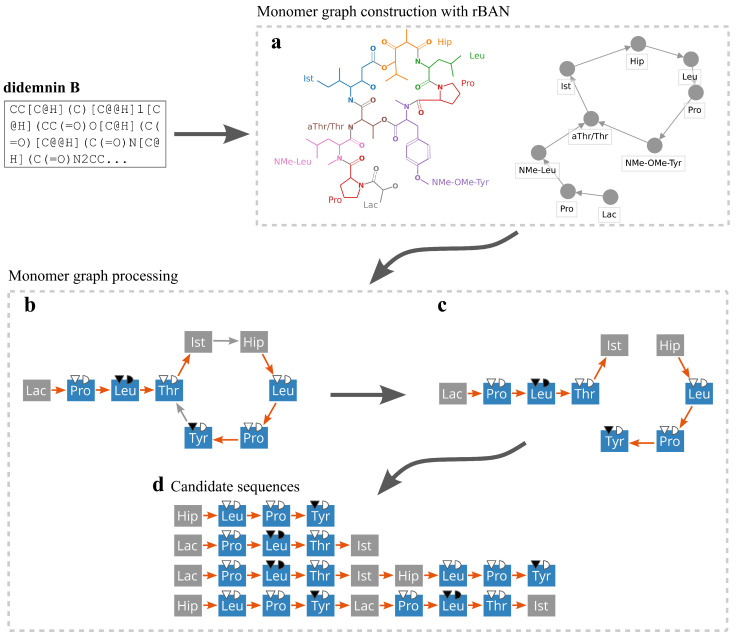
Linearization of didemnin B [48]. (**a**) The structure is analyzed with rBAN producing the monomer graph representation. (**b**) Edges are classified into backbone (orange) and tailoring (grey). Nodes are classified into supported by Nerpa (blue) and all others (grey). (**c**) All tailoring bonds are removed and (**d**) candidate linear representations of the peptide are generated. The (**a**) part of the figure was created using the rBAN web server at https://web.expasy.org/rban/ (accessed on 30 September 2021).

NRP processing normally starts with a database of chemical structures in the isomeric SMILES format and consists of several steps explained in detail below (Figure 1a,b and Figure 4). Non-isomeric SMILES are also supported, but their use may later lead to a less accurate matching of NRPs to predicted BGCs (Figure 1e). A user may also provide NRPs in a custom monomer graph format described in the Nerpa documentation. In this case, Nerpa skips the pre-processing and proceeds to the matching step (Figure 1e). The latter format could be useful for analysing compounds from Norine [30], since many of them lack SMILES representation.

#### 4.1.1. Monomer Graph Construction

Nerpa converts the input structures into monomer graphs using rBAN [33]. The monomer graph represents NRP structures as directed graphs with nodes representing monomers and edges representing bonds and heterocycles linking the monomers (Figure 4a,b). rBAN builds a monomer graph by breaking the structure according to a built-in set of fragmentation rules, and matches fragments to a database of known monomers. rBAN outputs the monomer graph and its mapping to the original atomic structure. The built-in rBAN database includes 909 monomers extracted from annotated compounds from the Norine database [30] and complemented with their common modifications. We further expanded this database by adding 35 in-house monomers. The full list of supported monomers is available from the Nerpa website.

#### 4.1.2. Monomer Post-Processing

Nerpa post-processes the rBAN output to infer monomer stereochemistry and recognize unidentified monomers. While rBAN ignores the stereochemistry, Nerpa inspects all chiral centers in the original chemical structure, allowing it to determine the stereoisomeric configuration (D-/L-) of the monomers. While rBAN performs the retro-biosynthesis of NRPs only, Nerpa supports NRP-polyketide hybrids [49]. rBAN ignores carbon-carbon bonds between NRP and polyketide monomers that result in unidentified monomers consisting of an amino acid attached to a polyketide chain. By inspecting the atomic structure of each unidentified monomer, Nerpa recognizes the chains and scans the remaining substructures against the database of known NRP monomers. Using this strategy Nerpa recovers correct NRP monomers that are not identified by rBAN.

#### 4.1.3. Linear Representation of Monomer Graphs

We classify edge annotations in the monomer graph into the backbone and tailoring classes. The backbone class consists of bond types that can be attributed to the activity of core NRPS modules. This class includes amide and double amide bonds and heterocycles such as thiazole, oxazole, and pyrimidine. All other bond types form the tailoring class. We also classify all graph nodes into Nerpa-supported and unsupported monomers. The former category contains monomers that can be predicted from a BGC (Figure 1c,d). The latter category contains all the rest monomers from the rBAN database.

Nerpa starts linearization of a monomer graph by removing all tailoring bonds (Figure 4). Then the tool independently processes all weakly connected graph components (Figure 4c,d). Components including less than a certain number of supported monomers (user-configurable, default is 2) are discarded. Nerpa proceeds with finding a Hamiltonian path or a Hamiltonian cycle within the component. In the former case, the path is considered as a candidate NRP monomer sequence. In the latter case, all possible linearizations starting from each node of the cycle are considered. Further, if the graph lacks cycles and includes at most three linear components, Nerpa considers all their permutations as additional candidate sequences. By permuting the components, Nerpa supports non-collinear NRPS assembly lines; by considering each component as a separate candidate sequence, Nerpa supports short iterative NRPs (Supplementary Figure S5).

### 4.2. Processing of Genome Sequences

Nerpa accepts genome sequences in the FASTA or GenBank format and processes them with the antiSMASH v5 genome mining pipeline [16]. Users can also directly provide the tool with antiSMASH outputs. Nerpa extracts NRPS genes, modules, and domains along with substrate specificity predictions from the antiSMASH output. Then, Nerpa combines specificity predictions into assembly lines, corrects misassembled BGCs, and handles non-collinear NRPS assembly lines (Figure 1c,d).

#### 4.2.1. BGC Monomers

Nerpa converts each NRPS module into a BGC monomer. The tool considers A domains, which define the core amino acids, and M and E domains, which determine whether the amino acid is methylated and its stereochemistry. A module may also contain a dual function C/E domain [8] that is treated as a regular C domain and an E domain in the previous module. Nerpa relies on the NRPSPredictor2 specificity predictions for A domains [17] generated in the antiSMASH v5 pipeline. NRPSPredictor2 reports a list of tentative amino acids complemented with two types of prediction scores. The first score assesses the sequence similarity between the ten-letter nonribosomal code of the domain and the database of known codes (the Stachelhaus score [5]). The second score relies on a Support Vector Machine (SVM) trained on previously annotated A domains (the SVM score [50]). Similar to the previous approaches [23,25], Nerpa ranks predicted amino acids by the mean of their Stachelhaus and SVM scores and selects the top one as the core amino acid of the BGC monomer.

#### 4.2.2. Monomer Strips

We define a *BGC monomer strip*, or simply a *strip*, of an NRPS gene as a sequence of BGC monomers corresponding to the gene’s modules. A strip may also include additional information, such as the presence of CS and TE domains in the gene (Figure 1c). CS and TE domains assist in determining the strip position inside the NRPS assembly line. Nerpa fixes the order of BGC monomers within a strip since the order of modules in a gene is always conserved during the NRP synthesis.

After forming the initial strips from all typical NRPS modules, Nerpa analyses seemingly deficient modules to infer additional strip variants. As previously described [51], a deficient module without an A domain may stutter, i.e., reuse an A domain of the previous typical module and thus recruit the same amino acid one or more times. A deficient module comprising a sole PCP domain and located last in an NRPS gene may iteratively reuse the entire gene [52]. Nerpa complements the initial strip of a gene having deficient module(s) with alternative strip variants to account for these events. The variants include up to three BGC monomer copies per each deficient module without an A domain and up to three copies of the entire strip per each deficient module with a sole PCP domain.

#### 4.2.3. BGC Splitting

Occasionally, antiSMASH v5 incorrectly identifies BGC boundaries in BGC-rich genome sequences and reports two or more adjacent BGCs as a single large cluster. Such errors are even present in manually curated datasets, for instance, the syringomycin BGC in MIBiG (BGC0000437) actually contains two clusters responsible for synthesis of syringomycin and syringopeptin (also available in MIBiG as a separate entry BGC0000438). Erroneously merged BGCs complicate downstream analysis and deteriorate Nerpa results. To address this problem, we process each multi-gene BGC with two simple heuristics.

First, Nerpa calculates the distances between adjacent NRPS genes in a BGC and if a distance exceeds a user-configurable threshold MAX_BGC_DIST (10,000 nucleotides by default) the BGC is split between the genes. Next, we check the consistency of CS and TE domains in the remaining BGCs. Usually, if a BGC contains a CS (TE) domain, this domain belongs to the cluster’s very first (last) gene. Nerpa splits BGCs before (after) genes containing a CS (TE) domain to meet this condition. At the same time, an inconsistent CS/TE domain location may also indicate a BGC with a non-collinear NRPS assembly line. To account for this possibility, Nerpa keeps the unsplit copies of such BGCs to be processed with the non-collinearity handling algorithm.

#### 4.2.4. Handling of NRPS Assembly Lines

The final step of the genome post-processing is the generation of a *BGC monomer sequence* or a set of plausible sequences reflecting the (unknown) NRPS assembly line of a BGC (Figure 1d). Under the collinear assembly line assumption, the BGC monomer sequence is simply a concatenation of all respective BGC monomer strips ordered the same as their corresponding NRPS genes in the BGC. However, the sequence generation may become extremely challenging when non-collinear NRPS assembly lines are taken into account. In this case, all possible permutations of the strips should be considered. The number of the permutations grows super-exponentially and the downstream processing becomes computationally prohibitive even for a relatively small number of genes.

Nerpa uses a heuristic approach to determine whether to apply the collinear or non-collinear NRPS assembly line for a given BGC and to effectively reduce the number of possible permutations in the latter case. The method analyses optional domains in the inward and outward (i.e., the first and the last) BGC monomer strips. In addition to CS and TE domains discussed above, Nerpa also considers communication-mediating (COM) domains [53]. COM domains consist of N-terminal and C-terminal subtypes named according to the domain location on the respective terminus of an NRPS gene. Two NRPS genes remotely located in a BGC could be linked in the NRPS assembly line thanks to the interaction between their C-terminal and N-terminal COM domains. However, currently it is impossible to computationally predict the proclivity of a C-terminal COM domain to a specific N-terminal COM domain. Nerpa classifies a sequence of BGC monomer strips as consistent if (i) CS domain (if present) is located in the first strip; (ii) TE domain (if present) is located in the last strip; (iii) the first strip lacks an N-terminal COM domain; and (iv) the last strip lacks a C-terminal COM domain.

Given a BGC, Nerpa first assumes the collinear NRPS assembly line and constructs the BGC monomer sequence accordingly. If the sequence is consistent, the processing is complete. If the inconsistency is caused solely by violation of conditions (i) or (ii), the tool puts the corresponding strip to the very beginning or ending of the sequence (Supplementary Figure S6). Otherwise, Nerpa considers all strip permutations resulting in consistent BGC monomer sequences. That is, the strips containing CS and TE domains (if present) are located at the beginning and end, and the rest of positions are subject to permutations. Additionally, a strip starting with N-terminal COM domain cannot be the first and a strip ending with C-terminal COM domain cannot be the last (Supplementary Figure S7).

### 4.3. Scoring of Monomer Sequences

The Nerpa scoring module takes as an input one or several possible NRP and BGC monomer sequences (Figure 1e). We perform the global alignment between all sequence pairs and report the best scoring pair as the most likely explanation of the NRP by the BGC. Without loss of generality, below we define the Nerpa score for a given global alignment of a monomer sequence pair. The optimal (best-scoring) global alignment for each pair is computed using the Needleman–Wunsch algorithm [32].

#### 4.3.1. General Notations

Let A be the alphabet of Nerpa-supported amino acids, ⌀ be a special sign designating any unsupported amino acid. Many distinct unsupported amino acids are possible, so we assume ⌀≠⌀. The extended alphabet of NRP/BGC monomer core amino acids is defined as $\bar{A}=A\cup \left\{⌀\right\}$. We use the notation $M^{NRP}=\left(a^{NRP},m^{NRP},e^{NRP}\right)$ to denote an NRP monomer with a core amino acid $a^{NRP}\in \bar{A}$, $m^{NRP}=1$ if the amino acid is methylated and −1 otherwise, $e^{NRP}=1$ if the amino acid is in the D-configuration and −1 if it is in the L-configuration; $e^{NRP}$ could also be equal to 0 if it is impossible to enquire amino acid stereochemistry from the NRP structure or if it is irrelevant, e.g., for glycine, which is a non-chiral amino acid. Analogously, we designate a BGC monomer as $M^{BGC}=\left(s,a^{BGC},m^{BGC},e^{BGC}\right)$, where $a^{BGC}\in A$, $m^{BGC},e^{BGC}\in \left\{-1,1\right\}$ and s∈[0⋯100] corresponds to the specificity prediction score. Here s=0 is a special value corresponding to a completely unreliable prediction and s>0 is a software-generated score with s=100 being the most trustworthy prediction. We also define an undefined BGC monomer as ${\bar{M}}^{BGC}=\left(0,⌀,0,0\right)$, where $m^{BGC}=0$ ($e^{BGC}=0$) indicates undefined genomic prediction of the methylation state (stereochemistry). For convenience, we further use $\emptyset^{NRP}=\left(-,-,-\right)$ and $\emptyset^{BGC}=\left(-,-,-,-\right)$ to designate the absence of a monomer in a global sequence alignment with indels.

A string $NRP=M_{1}^{NRP}M_{2}^{NRP}\cdots M_{n}^{NRP}$ is an NRP monomer sequence, its length is |NRP|=n, *i*th monomer is $NRP\left[i\right]=M_{i}^{NRP}$. Similarly, $BGC=M_{1}^{BGC}M_{2}^{BGC}\cdots M_{m}^{BGC}$ is a BGC monomer sequence of length *m*. A global alignment of NRP and BGC is a pair $\left(NRP^{\prime },BGC^{\prime }\right)$ such that $|NRP^{\prime }|=|BGC^{\prime }|=l$, n+m≥l≥max(n,m), NRP (BGC) is a subsequence of $NRP^{\prime }$ ($BGC^{\prime }$), the supersequence may additionally contain only monomers equal to $\emptyset^{NRP}$ ($\emptyset^{BGC}$) and if $NRP^{\prime }\left[i\right]=\emptyset^{NRP}$ then $BGC^{\prime }\left[i\right]\neq \emptyset^{BGC}$ and vice versa.

#### 4.3.2. Nerpa Score Summary

Given an NRP monomer sequence NRP, a BGC monomer sequence BGC and their global alignment $\left(NRP^{\prime },BGC^{\prime }\right)$ we estimate the probability $P(NRP^{\prime }|BGC^{\prime })$ of the NRPS assembly line encoded in BGC to synthesize NRP as prescribed in the alignment. That is, a BGC module corresponding to $BGC^{\prime }\left[i\right]$ is responsible for incorporating a residue $NRP^{\prime }\left[i\right]$ into the NRP structure. We also formulate a null hypothesis that NRP is synthesised by an undefined BGC monomer sequence NULL such that |NULL|=|NRP| and $NULL\left[i\right]={\bar{M}}^{BGC}\;\forall i\in \left[1\cdots n\right]$. We further compute the null hypothesis probability P(NRP|NULL) and define the Nerpa score as a log odds ratio of the two probabilities

(1) $$Score\left(NRP^{\prime },BGC^{\prime }\right)=\log\frac{P(NRP^{\prime }|BGC^{\prime })}{P(NRP|NULL)}$$

We assume the independence of the aligned monomer pairs and rewrite Equation (1) as:

(2) $$\begin{array}{cc} Score(NRP^{\prime },BGC^{\prime }) & =\log\frac{\prod_{i=1}^{l}P\left(NRP^{\prime }\left[i\right]|BGC^{\prime }\left[i\right]\right)}{\prod_{i=1}^{n}P\left(NRP\left[i\right]|NULL\left[i\right]\right)}\\ \; & =\sum_{i=1}^{l}\log P\left(NRP^{\prime }\left[i\right]|BGC^{\prime }\left[i\right]\right)-\sum_{i=1}^{n}\log P\left(NRP\left[i\right]|NULL\left[i\right]\right)\\ \; & =\sum_{i=1}^{l}\log P\left(a_{i}^{NRP},m_{i}^{NRP},e_{i}^{NRP}|s_{i},a_{i}^{BGC},m_{i}^{BGC},e_{i}^{BGC}\right)\\ \; & -\sum_{i=1}^{n}\log P\left(a_{i}^{NRP},m_{i}^{NRP},e_{i}^{NRP}|0,⌀,0,0\right). \end{array}$$

For simplicity, we also assume that the matches of core amino acids, their methylations and stereochemistry affect the total probability independently. Therefore, Equation (2) is a sum of three components:

$$Score\left(NRP^{\prime },BGC^{\prime }\right)=Score^{A}\left(NRP^{\prime },BGC^{\prime }\right)+Score^{M}\left(NRP^{\prime },BGC^{\prime }\right)+Score^{E}\left(NRP^{\prime },BGC^{\prime }\right),$$

where

(3) $$\begin{array}{cc} Score^{A}\left(NRP^{\prime },BGC^{\prime }\right) & =\sum_{i=1}^{l}\log P\left(a_{i}^{NRP}|s_{i},a_{i}^{BGC}\right)-\sum_{i=1}^{n}\log P\left(a_{i}^{NRP}|0,⌀\right)\\ Score^{M}\left(NRP^{\prime },BGC^{\prime }\right) & =\sum_{i=1}^{l}\log P\left(m_{i}^{NRP}|m_{i}^{BGC}\right)-\sum_{i=1}^{n}\log P\left(m_{i}^{NRP}|0\right)\\ Score^{E}\left(NRP^{\prime },BGC^{\prime }\right) & =\sum_{i=1}^{l}\log P\left(e_{i}^{NRP}|e_{i}^{BGC}\right)-\sum_{i=1}^{n}\log P\left(e_{i}^{NRP}|0\right). \end{array}$$

#### 4.3.3. Scoring Matches and Mismatches

Consider an alignment without indels. We compute the probability of a BGC module with substrate prediction $a^{BGC}$ and specificity score *s* to synthesize a core NRP monomer amino acid $a^{NRP}$ as

(4) $$P\left(a^{NRP}|s,a^{BGC}\right)=\left\{\begin{array}{cc} P_{match}^{A}\left(s\right) & \;\mathrm{if}\;a^{NRP}=a^{BGC}\\ \left(1-P_{match}^{A}\left(s\right)\right)\cdot \frac{P^{A}\left(a^{NRP}\right)}{1-P^{A}\left(a^{BGC}\right)} & \;\mathrm{if}\;a^{NRP}\neq a^{BGC}, \end{array}\right.$$

where $P^{A}\left(a\right)$ determines how likely amino acid *a* may appear in an NRP by random chance, that is, how frequent *a* is comparing to all other NRP amino acids, $\sum_{a\in \bar{A}}P^{A}\left(a\right)=1$. For the null hypothesis component of $Score^{A}$ we thus obtain

$$P\left(a^{NRP}|0,⌀\right)=\left(1-P_{match}^{a}\left(0\right)\right)\cdot \frac{P^{A}\left(a^{NRP}\right)}{1-P^{A}\left(⌀\right)}=\frac{P^{A}\left(a^{NRP}\right)}{1-P^{A}\left(⌀\right)},$$

since $P_{match}^{a}\left(0\right)=0$ by convention.

We simplified Equation (4) to keep the number of Nerpa parameters reasonable. For instance, here we assume the match probability depends only on the specificity score but not the underlying amino acid. In practice, the probabilities vary for different amino acids but the size of $\bar{A}$ is too large to account for all possible options. In contrast, *m* and *e* monomer components have at most three different states (−1,0,1), so we defined the corresponding probabilities in $Score^{M}$ and $Score^{E}$ more flexibly than in (4). Below is the formula for the *m* component of the score. We compute the *e* counterpart similarly with the only difference being that $e^{NRP}$ could be 0. This case is uninformative regardless of the $e^{BGC}$ value, so we define $P(0|e^{BGC})=1$ resulting in no contribution to the log score.

(5) $$P\left(m^{NRP}|m^{BGC}\right)=\left\{\begin{array}{cc} P_{match}^{M}\left(m\right) & \;\mathrm{if}\;m^{NRP}=m^{BGC}=m\in \left\{-1,1\right\}\\ P_{mismatch}^{M}\left(m\right) & \;\mathrm{if}\;m^{NRP}=-m^{BGC}=m\in \left\{-1,1\right\}\\ P^{M}\left(m^{NRP}\right) & \;\mathrm{if}\;m^{BGC}=0,m^{NRP}\in \left\{-1,1\right\}, \end{array}\right.$$

where $P^{M}\left(m\right)$ determines how often NRP residues are methylated (m=1) or not methylated (m=−1). Thus, the null hypothesis component of $Score^{M}$ is $P^{M}\left(m^{NRP}\right)$.

#### 4.3.4. Scoring Indels

By the null hypothesis formulation, indels may only occur in the $P(NRP^{\prime }|BGC^{\prime })$ component of the Nerpa score (1). For a monomer pair $\left(M^{NRP},M^{BGC}\right)$ in the alignment, we compute the probability of an insertion, that is, $M^{BGC}={⌀}^{BGC}$, as

$$P\left(M^{NRP}|{⌀}^{BGC}\right)=P\left(a,m,e|{⌀}^{BGC}\right)=P_{insertion}\cdot P^{A}\left(a\right)\cdot P^{M}\left(m\right)\cdot P^{E}\left(e\right),$$

where $P_{insertion}$ is the insertion probability and $P^{A}$, $P^{M}$, $P^{E}$ have the same value and meaning as in Equations (4) and (5). In contrast, we assume deletions are independent from specific amino acids, methylation and stereochemistry and may occur by random chance with the uniform probability $P\left({⌀}^{NRP}|M^{BGC}\right)=P_{deletion}$.

#### 4.3.5. Learning Nerpa Parameters

The Nerpa scoring relies on two major groups of parameters. The first group consists of $P^{A}$, $P^{M}$, and $P^{E}$, describing average frequencies of core amino acids, methylations and stereochemistry configurations in NRP monomers, respectively. All curated and putative NRP structures available in the SMILES format in the latest version of Norine [30] were used to estimate these parameters. The training dataset #1 was comprised of 625 structures grouped in 182 NRP families.

The training of the remaining parameters requires known BGC-NRP alignments. For this purpose, we formed a dataset of 64 representative NRPS BGCs from MIBiG v2 [28]. For each entry, we extracted the corresponding monomer sequences and manually constructed their global alignment based on the referenced publications. The resulting training dataset #2 comprised 607 monomer pairs including matches, mismatches, and indels (Supplementary File S2).

Before the training, the real specificity score scale [1⋯100] was discretized into five score levels to reduce the number of Nerpa parameters. We estimated parameters using frequencies of the corresponding events. For example, $P_{mismatch}^{E}\left(-1,1\right)$ is the number of alignment sites having the NRP monomer in the L-configuration ($e^{NRP}=-1$) and the BGC monomer suggesting epimerization ($e^{BGC}=1$) to the total number of non-indel sites where the NRP monomer stereochemistry is determined ($e^{NRP}\neq 0$).

We generated 100 equally-sized bootstrap samples from the training dataset #2 and used them for parameter learning. Supplementary Figure S8 shows parameter distributions; the final parameter values for both groups are in Supplementary Tables S1 and S2. All parameters are also available online in the Nerpa configuration files. A user may form a custom dataset of BGC-NRP alignments and re-calculate the parameters using training scripts from the tool repository.

### 4.4. Reporting of Results

In the final step of the pipeline, Nerpa summarizes scoring results for all inputs (Figure 1e). First, BGC-NRP matches having a score below a user-specified threshold MIN_SCORE (6.0 by default) are discarded. Next, the remaining hits are grouped by compound and by genome and reported together with the underlying alignments. We also compose a combined report with the most promising matches overall. A simple list of the best scoring hits could be misleading here. Sometimes, a large dataset includes a few peculiar BGCs (NRPs) producing high-scoring matches with many distinct compounds (genomes). As a result, the list becomes uninformative since it is dominated by these few BGCs (NRPs). To address this issue, Nerpa includes a BGC-NRP match in the combined report only if the corresponding NRP is among the best hits per the matching BGC, and the BGC is among the best hits per the NRP simultaneously.

### 4.5. Software Implementation

Nerpa is implemented in C++ and Python v3. The NRP structure decomposition and linearization relies on rBAN [33], the RDKit framework [54] and the NetworkX library for graph manipulation [55]. Genome mining is made with the antiSMASH v5 pipeline [16] utilizing NRPSpredictor2 [17] for the substrate specificity prediction. The Nerpa combined report is in a plain tab-separated format. The detailed per NRP and per genome reports are in a custom text format depicting linearized NRP and BGC monomer sequences and their Nerpa alignment along with the scores for each monomer pair.

## Figures and Tables

**Table 1:** Running time and memory usage of GARLIC and Nerpa in the search of the RefSeq genomes against pNRPdb. The resource consumption is given separately for each step of the GARLIC (GRAPE, PRISM, and GARLIC) and Nerpa (rBAN, antiSMASH, and Nerpa) pipeline and in total. The first row in each group shows means and standard deviations in the five independent runs, 100 genomes each. The second row gives estimated (∼) and exact values for the full dataset processing (13,399 genomes). The structure parsing step was done once for the entire database (8368 structures). All benchmarking was done on a server node with 16 Intel Xeon X7560 2.27 GHz CPUs.

# Genomes	Structure Parsing		Genome Mining		Matching		The Full Pipeline	
	GRAPE	rBAN	PRISM	antiSMASH	GARLIC	Nerpa	GARLIC	Nerpa
**Running Time (d-h:m)**								
100	3:41	0:17	3:52 ± 1:41	0:05 ± 0:01	13:00 ± 1:09	0:57 ± 1:24	20:33 ± 2:41	1:19 ± 1:25
13,399			∼21-14:08	23:36	∼72-14:00	1-16:14	∼94-04:08	2-16:07
**Peak RAM Consumption (GB)**								
100	16.3	13.1	40.2 ± 1.2	3.6 ± 0.4	2.5 ± 0.9	0.6 ± 0.4	40.2 ± 1.2	13.1
13,399			∼40.2	4.8	∼2.5	18.6	∼40.2	18.6

**Table 2:** Selected BGC-NRP pairs from the RefSeq experiment. The first three columns are from the chemical structures input, the next two are from the genomic counterpart. Compound producer and reference are given according to the pNRPdb metadata. The score stands for the Nerpa score. The last column contains IDs in the MIBiG or NCBI protein database if similar NRPS genes were found, gene names are given in parenthesis. Microcystin-LR’ is for <L-MeSer7>microcystin-LR.

ID pNRPdb	Compound	Producer	ID GenBank	Species	Score	Known Analog
NPA011095	Ohmyungsamycin A	*Streptomyces* sp. [41]	GCA_013364095.1	*S. harbinensis*	27.31	QGA70148.1 (ohmA)
NPA014983	Microcystin-LR’	*Microcystis* sp. [42]	GCA_000010625.1	*M. aeruginosa*	13.88	BGC0001017 (mcyA,B,C,E)
NPA002702	Ngercheumicin F	*Photobacterium* sp. [43]	GCA_000695255.1	*P. galatheae*	12.76	**—**
NPA024438	Stephensiolide F	*Serratia* sp. [40]	GCA_017309605.1	*S. ureilytica*	11.69	ATD12179.1 (sphA)

## Data Availability

Nerpa source code is available from GitHub at http://github.com/ablab/nerpa (accessed on 30 September 2021). The training dataset and results of the RefSeq experiment are available in the Supplementary Materials. The MS/MS data used in this study were deposited in the GNPS MassIVE repository as MSV000086428 (accessed on 30 September 2021). The pNRPdb database, metadata of the MIBiG_NRP_ and RefSeq experiments, and Nerpa-preprocessed source data: pNRPdb structures, MIBiG_NRP_ and RefSeq genomes are openly available in Zenodo at https://doi.org/10.5281/zenodo.5503984 (accessed on 30 September 2021). Dereplicator annotations of ngercheumicin spectra in MSV000086428 can be accessed at https://gnps.ucsd.edu/ProteoSAFe/result.jsp?task=e6d6116ce525405ba46ec61dbdef96ef&view=view_significant_unique#%7B%22main.Name_input%22%3A%22nger%22%7D (accessed on 30 September 2021), Dereplicator+ annotations at https://gnps.ucsd.edu/ProteoSAFe/result.jsp?task=069cd972b8eb460e892e9023ff339a80&view=view_significant_unique#%7B%22main.Name_input%22%3A%22nger%22%7D (accessed on 30 September 2021), and the ngercheumicin molecular network at https://gnps.ucsd.edu/ProteoSAFe/result.jsp?view=network_displayer&componentindex=5&task=786ef25ff82d4872951fe7d26a72c4f6&show=true (accessed on 30 September 2021).

## Supplementary Materials

The following are available online at https://www.mdpi.com/article/10.3390/metabo11100693/s1, Figure S1: Contribution of source databases to pNRPdb (8368 compounds), Figure S2: Distribution of pNRPdb compounds producers, Figure S3: Taxonomic group distribution of 13,399 reference and representative NCBI RefSeq [37] bacterial genomes visualized with Krona [56], Figure S4: Ngercheumicin structure, putative biosynthetic gene cluster and their Nerpa alignment, Figure S5: Linearization of iterative nonribosomal peptide bacillibactin [57], Figure S6: Processing of colistin A biosynthetic gene cluster (BGC) from *Paenibacillus alvei* (MIBiG BGC0001192) [58], Figure S7: Processing of amychelin biosynthetic gene cluster (BGC) from *Streptomyces* sp. AA4 (MIBiG BGC0000300) [59], Figure S8: Distributions of the Nerpa parameters estimated from 100 bootstrap samples from the training dataset, Table S1: Core amino acids supported by Nerpa along with their PubChem CIDs [60] and scores, Table S2: Nerpa scoring parameters, Note: Inspection of GARLIC false positive identifications (Supplementary_Material.pdf), File S1: 117 BGCs from the RefSeq representative bacterial genomes linked by Nerpa to their putative products (Supplementary_File_S1.tsv), File S2: Nerpa training dataset with the global alignments of 64 BGC-NRP pairs from MIBiG (Supplementary_File_S2.tsv).

## Abbreviations

The following abbreviations are used in this manuscript:

BGC	biosynthetic gene cluster
FDR	false discovery rate
GB	gigabyte
GNPS	Global Natural Product Social molecular networking
MS/MS	tandem mass spectra
NCBI	National Center for Biotechnology Information
NRP	nonribosomal peptide
NRPS	nonribosomal peptide synthetase
RAM	random access memory (computer memory)
RefSeq	NCBI Reference Sequence Database
RiPP	ribosomally synthesized and post-translationally modified peptide
SD	standard deviation

## Appendix A. Running Commands

In this appendix, we provide running commands of GARLIC, Nerpa, and their pipeline components used in benchmarking.

PRISM version 2.1.5, downloaded from https://github.com/magarveylab/prism-releases/releases/download/v2.1.5/prism.jar (accessed on 30 September 2021)
  java -jar <PATH_TO_PRISM_JAR> -a -p \
      -f <PATH_TO_GENOME_FASTA> -w 10000 \
      -tt -o <OUTPUT_DIR> -r <PATH_TO_WEBCONTENT>
GRAPE version 2.9.1, downloaded from https://github.com/magarveylab/grape-release/archive/refs/tags/2.9.1.tar.gz (accessed on 30 September 2021)
  java -jar <PATH_TO_GRAPE_JAR> -s <SMILES_STRING> \
      -img -json -txt -o <OUTPUT_DIR>
GARLIC version 1.0.2, downloaded from https://github.com/magarveylab/garlic-release/archive/refs/tags/1.0.2.tar.gz (accessed on 30 September 2021)
  java -jar <PATH_TO_GARLIC_JAR> -q <PATH_TO_PRISM_OUT> \
      -a <PATH_TO_GRAPE_OUT> -o <OUTPUT_DIR>
antiSMASH version 5.2.0, downloaded from https://dl.secondarymetabolites.org/releases/5.2.0/antismash-5.2.0.tar.gz (accessed on 30 September 2021)
  run_antismash.py <PATH_TO_GENOME_FASTA> \
      --genefinding-tool prodigal --minimal \
      --skip-zip-file --enable-nrps-pks \
      --output-dir <OUTPUT_DIR>
rBAN version 1.0, downloaded from https://bitbucket.org/sib-pig/rban/downloads/rBAN-1.0.jar (accessed on 30 September 2021)
  java -jar <PATH_TO_RBAN_JAR> \
      -monomersDB <PATH_TO_MONOMERS> \
      -inputFile <PATH_TO_INPUT_JSON> \
      -outputFileName <OUTPUT_FILE_NAME> \
      -outputFolder <OUTPUT_DIR>
Nerpa version 1.0.0, downloaded from https://github.com/ablab/nerpa/releases/tag/v1.0.0 (accessed on 30 September 2021)
  nerpa.py -a <PATH_TO_ANTISMASH_OUTPUTS> \
      --smiles-tsv <PATH_TO_STRUCTURES> \
      --col-smiles <COL_SMILES> --col-id <COL_ID> \
      --sep <SEP> --process-hybrids -o <OUTPUT_DIR>

## Appendix B. MS/MS Experiment Details

### Appendix B.1. Microorganism Culturing

*Photobacterium galatheae* DSM 100496 [44] was purchased from the Leibniz-Institut DSMZ-Deutsche Sammlung von Mikroorganismen und Zellkulturen GmbH. Media 514 and 830 (with 3% NaCl) were prepared as recommended by the strain provider (https://www.dsmz.de/collection/catalogue/microorganisms/culture-technology/list-of-media-for-microorganisms) (accessed on 30 September 2021). The microorganism was initially grown in 50 mL Erlenmeyer flasks containing 25 mL of media in a rotary shaker (MaxQ 4450, Thermo Scientific, Waltham, MA, USA) at 200 rpm with controlled temperature 28 or 30 °C for two days. A 100 uL microbial inoculum from a two-days culture was transferred into 900 uL culture in 96-deepwell plates and cultured at 28 and 30 °C during six days at static conditions.

### Appendix B.2. Extraction of Metabolites

The microbial cultures were submitted to three freeze-thaw cycles of 10 min each. After that, two methods of extraction were performed. One method consisted of directly adding methanol, followed by sonication for 15 min (Branson 5510, Marshall Scientific, Hampton, NH, USA), centrifugation for 15 min at 2000 rpm (865× *g*) (Sorvall Legend RT, Marshall Scientific, Hampton, NH, USA), transferring of supernatant to a clean 96-well plate and dried out in Centrifugal Vacuum Concentrator, Centrivap (Labconco, Kansas City, MO, USA). A second method consisted in Solid Phase Extraction (Hypersep C18 50 mg/1 mL, Thermo Scientific, Waltham, MA, USA). Extract was recovered with methanol, dried out under vacuum and prepared for LC-MS.

### Appendix B.3. Sample Preparation and LC-MS/MS Conditions

Samples were resuspended with 200 uL of 80% methanol containing 1uM amitriptyline as internal standard and LC-MS/MS analysis was performed in an UltiMate 3000 UPLC system (Thermo Scientific, Waltham, MA, USA) using a Kinetex 1.7 μm C18 reversed phase UHPLC column (50 × 2.1 mm) and Maxis-II Q-TOF mass spectrometer (Bruker Daltonics, Billerica, MA, USA) equipped with ESI source. The column was equilibrated with 5% solvent B (LC-MS grade acetonitrile, 0.1% formic acid) for 1 min, followed by a linear gradient from 5% B to 100% B in 8 min, held at 100% B for 2 min. Then, 100–5% B in 0.5 min and maintained at 5% B for 2.5 min at a flow rate of 0.5 mL/min throughout the run. MS spectra were acquired in positive ion mode in the range of 100–2000 *m/z*. A mixture of 10 mg/mL of each sulfamethazine, sulfamethizole, sulfachloropyridazine, sulfadimethoxine, amitriptyline, and coumarin was run after every 48 injections for quality control. An external calibration with ESI-Low Concentration Tuning Mix (*m/z* 118.086255; 322.048121; 622.028960; 922.009798; 1221.990637; 1521.971475; 1821.952313) (Agilent Technologies, Santa Clara, CA, USA) was performed prior to data collection. An internal calibrant Hexakis (1H,1H,2H-perfluoroethoxy) phosphazene (CAS 186817-57-2) was used throughout the runs. The capillary voltage of 4500 V, nebulizer gas pressure (nitrogen) of 2 bar, ion source temperature of 200 °C, dry gas flow of 9 L/min source temperature, spectral rate of 3 Hz for MS1 and 10 Hz for MS2 was used. For acquiring MS/MS fragmentation, the 6 most intense ions per MS1 were selected, MS/MS active exclusion parameter was enabled, set to 2 and to release after 30 s, precursor ion was reconsidered for MS/MS if current intensity/previous intensity ratio >2. Advanced stepping function was used to fragment ions according to the Table A1 settings. Used CID energies are specified in Table A2. The mass of the internal calibrant was excluded from the MS/MS list using a mass range of *m/z* 621.5–623.0.

**Table A1:** Instrument settings for data-dependent acquisition of samples.

Time	Collision RF	Transfer Time	Collision
0	450.0	70.0	125
25	550.0	75.0	100
50	800.0	90.0	100
75	1100.0	95.0	75

**Table A2:** CID energies for MS/MS data acquisition.

Type	Mass	Width	Collision	Charge State
Base	100.00	4.00	22.00	1
Base	100.00	4.00	18.00	2
Base	300.00	5.00	27.00	1
Base	300.00	5.00	22.00	2
Base	500.00	6.00	35.00	1
Base	500.00	6.00	30.00	2
Base	1000.00	8.00	45.00	1
Base	1000.00	8.00	35.00	2
Base	2000.00	10.00	50.00	1
Base	2000.00	10.00	50.00	2
