# Supplementary material for: Nerpa: A Tool for Discovering Biosynthetic Gene Clusters of Bacterial Nonribosomal Peptides
Source: Metabolites. 2021 Oct 11;11(10):693. doi: 10.3390/metabo11100693 (PMC8541647; doi:10.3390/metabo11100693)
Supplement: Supplementary file 1 [file metabolites-11-00693-s001.zip › supplement-metabolites-1399960/Supplementary_Material.pdf]

Supplementary Material for "Nerpa: A Tool for Discovering Biosynthetic Gene Clusters of Bacterial Nonribosomal Peptides"

Supplementary Figures

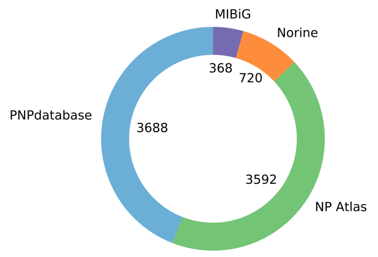

Supplementary Figure S1: Contribution of source databases to pNRPdb (8,368 compounds).

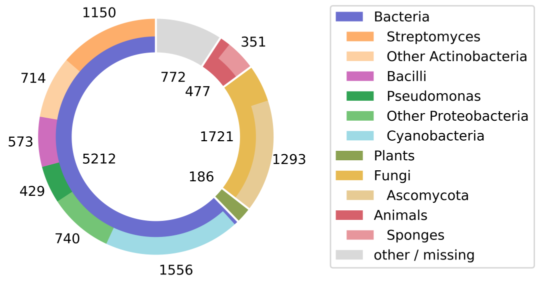

Supplementary Figure S2: Distribution of pNRPdb compounds producers. The outer circle highlights the most abundant taxonomic groups within the top taxonomic levels represented by the inner segments.

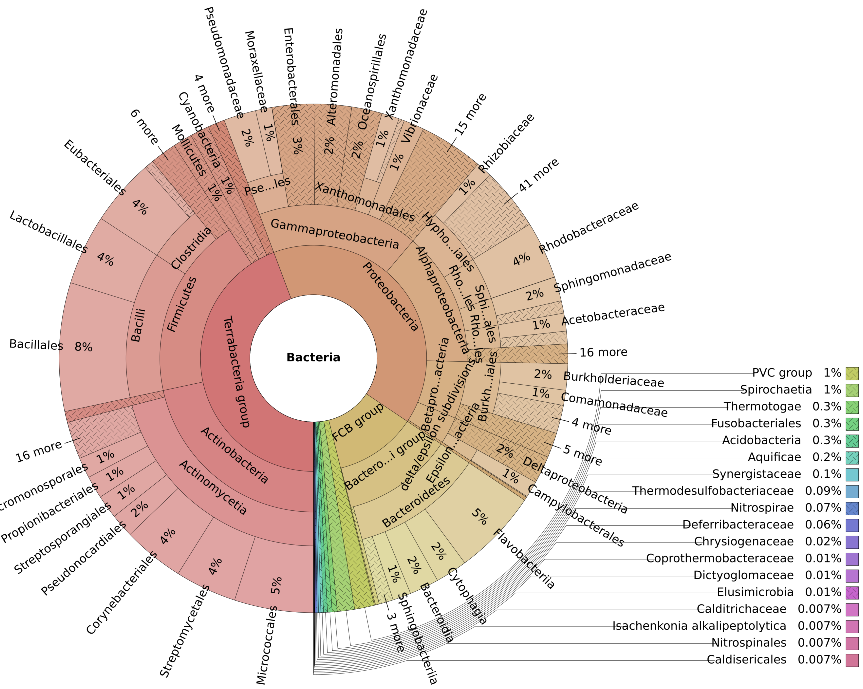

Supplementary Figure S3: Taxonomic group distribution of 13,399 reference and representative NCBI RefSeq [1] bacterial genomes visualized with Krona [2].

Genome: *Photobacterium galathea* (GCA_000695255.1)

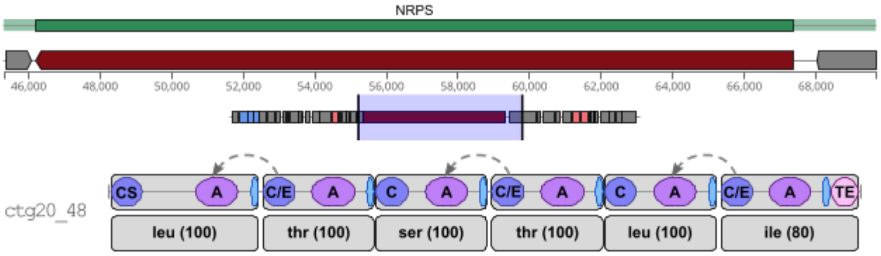

Compound: Ngercheumicin F (NPA002702)

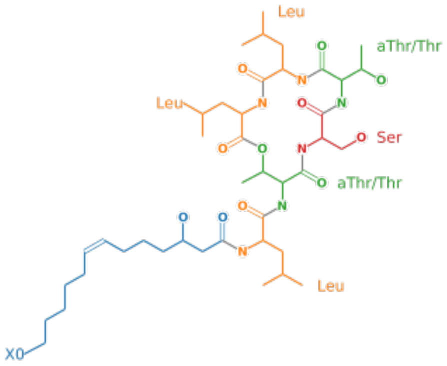

Nerpa alignment:

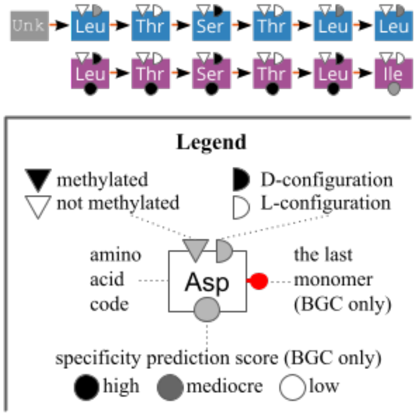

Supplementary Figure S4: Ngercheumicin structure, putative biosynthetic gene cluster and their Nerpa alignment. Dual function C/E domains are responsible for the epimerization of amino acids retained by the preceding modules as indicated by the grey dashed arrows.

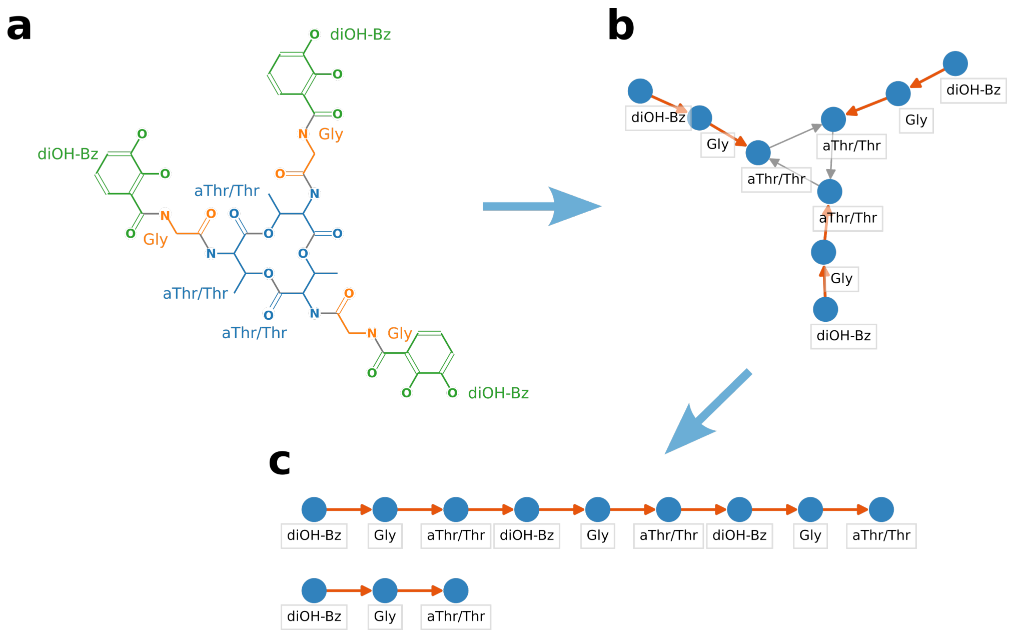

Supplementary Figure S5: Linearization of iterative nonribosomal peptide bacillibactin [3]. (a) rBAN annotates the structure into the monomer graph. (b) Nerpa classifies edges into backbone (orange) and tailoring (grey) and (c) removes tailoring bonds to generate linear representations of the peptide.

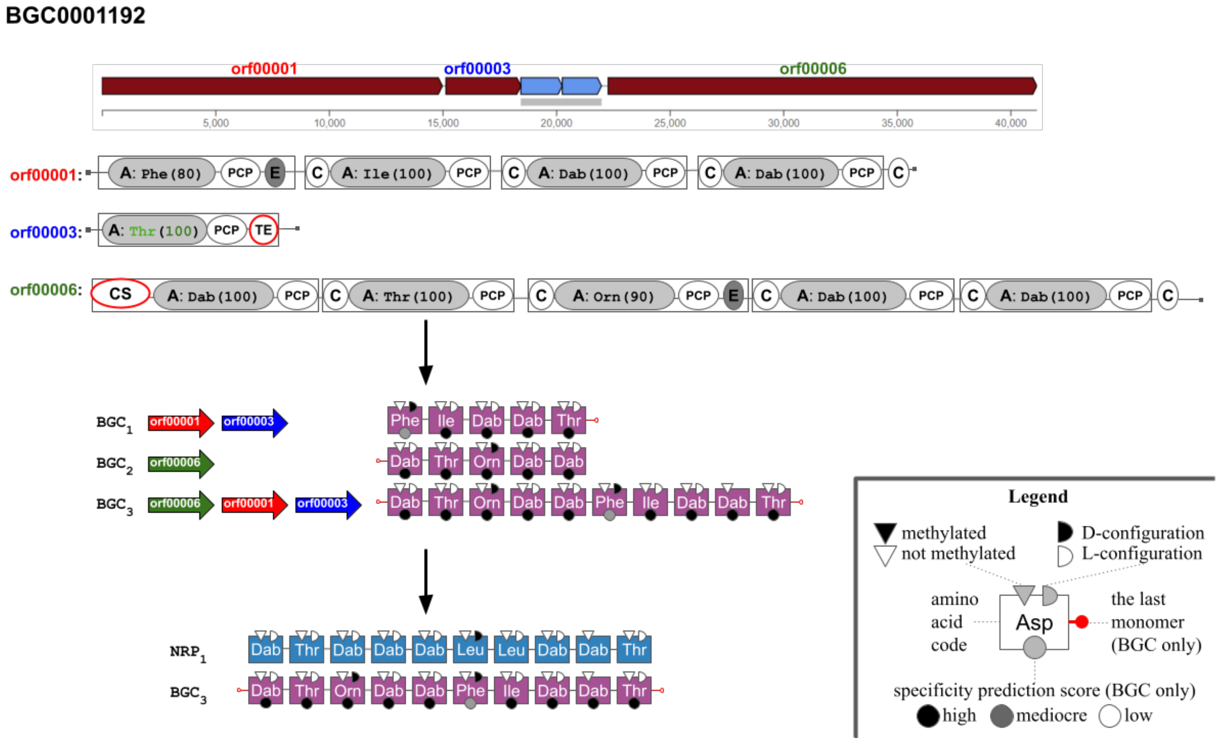

Supplementary Figure S6: Processing of colistin A biosynthetic gene cluster (BGC) from *Paenibacillus alvei* (MIBiG BGC0001192) [4]. AntiSMASH predicted three NRPS genes in the BGC: orf00001 (pmxA), orf00003 (pmxB), and orf00006 (pmxE). The middle gene (orf00003) contains a TE domain, so Nerpa considers two possible scenarios. Either the BGC should be split into two distinct BGCs by the TE domain (BGC_1 and BGC_2), or the BGC has a non-collinear NRPS assembly line and its genes should be reordered (BGC_3). In the latter case, Nerpa unequivocally derives the order of the genes by placing TE-containing orf00003 last and CS-containing orf00006 first. The matching with the NRP monomer sequence of colistin A supports the BGC_3 scenario. As a result, Nerpa correctly reveals the ground truth gene order of the colistin A biosynthetic pathway (pmxE-pmxA-pmxB) [4].

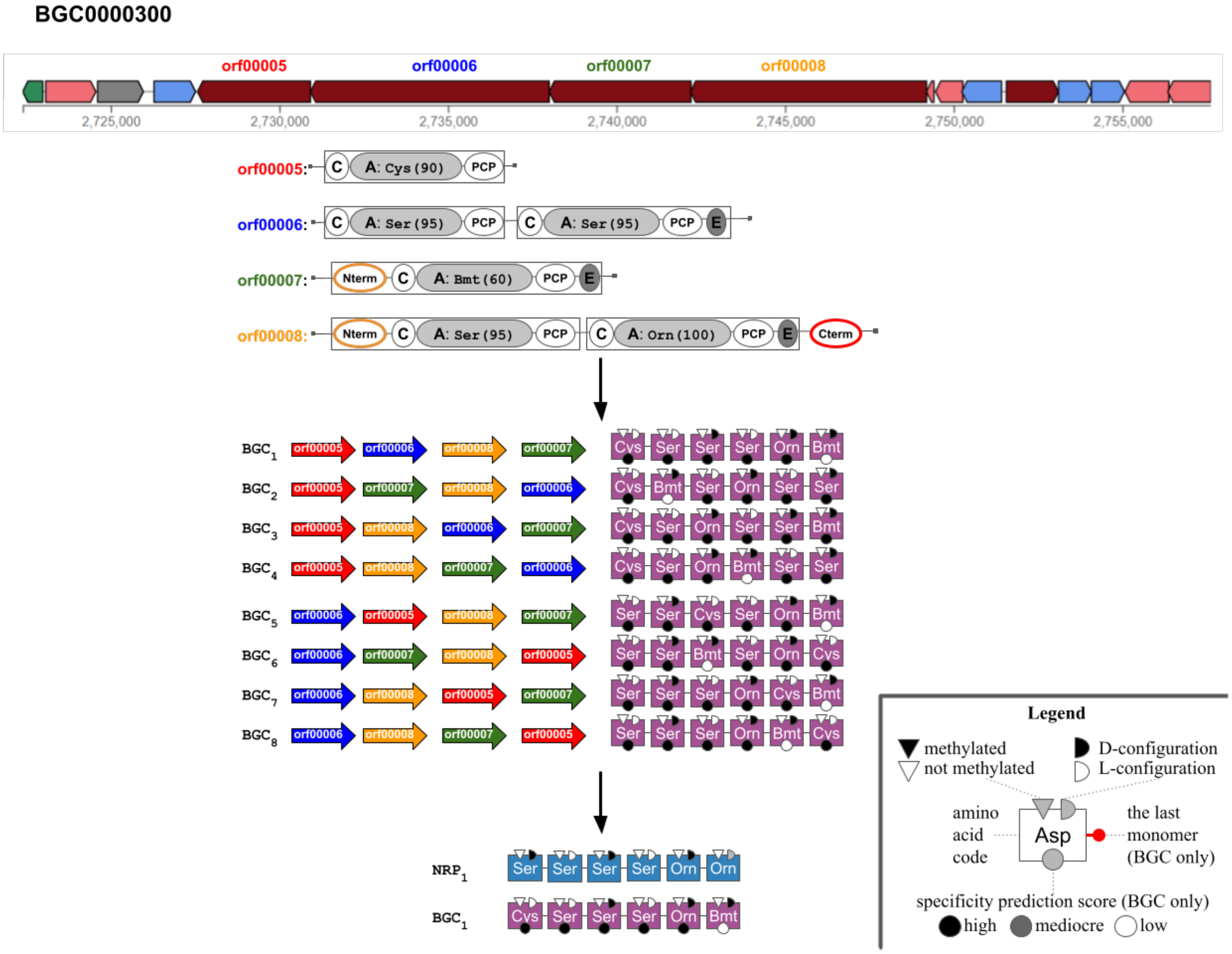

Supplementary Figure S7: Processing of amychelin biosynthetic gene cluster (BGC) from *Streptomyces* sp. AA4 (MIBiG BGC0000300) [5]. AntiSMASH predicted four NRPS genes on the reverse strand of the BGC: orf00005 (amcD), orf00006 (amcE), orf00007 (amcF), and orf00008 (amcG). The first gene (orf00008) starts with a C-terminal COM domain, suggesting a non-collinear NRPS assembly line for the BGC. Nerpa considers four-gene permutations that are COM domain-consistent, i.e., do not start with C-terminal and do not end with N-terminal. Thus, orf00008 cannot be the first and the last gene in the line, and orf00007 cannot be the first. As a result, Nerpa considers $2^3 = 8$ possible permutations, which is three times lower than $4! = 24$, the total number of all four-gene permutations. The matching with the amychelin NRP monomer sequence supports the BGC_1 scenario, corresponding to the correct gene order in the biosynthetic pathway (amcG-amcF-amcD-amcE) [5]

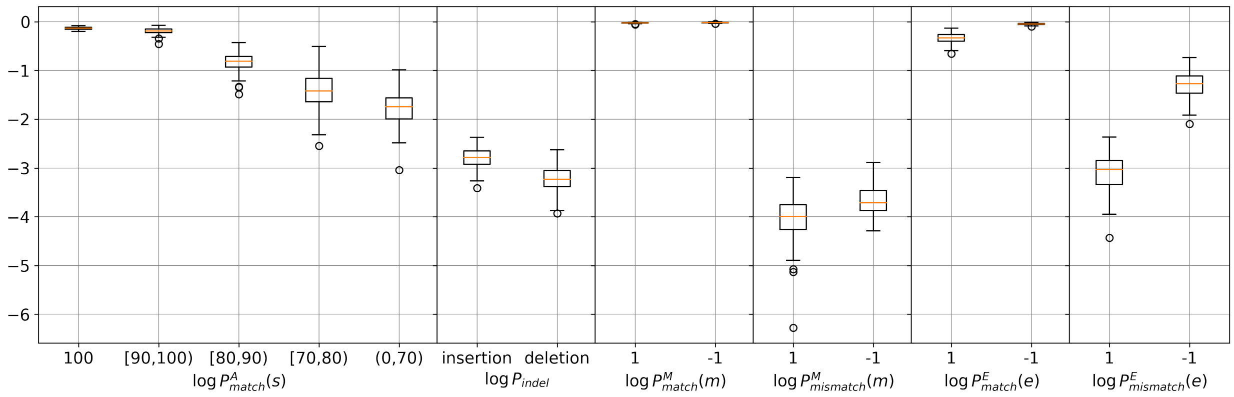

Supplementary Figure S8: Distributions of the Nerpa parameters estimated from 100 bootstrap samples from the training dataset.

Supplementary Tables

#	Code	Name	PubChem CID	$\log P^A(a^{NRP})$
1	leu	leucine	857	-2.28
2	val	valine	1182	-2.35
3	pro	proline	614	-2.43
4	ala	alanine	602	-2.52
5	phe	phenylalanine	994	-2.95
6	thr	threonine	205	-2.99
7	ser	serine	617	-3.02
8	gly	glycine	750	-3.07
9	ile	isoleucine	791	-3.09
10	gln	glutamine	738	-3.15
11	hpg	4-hydroxy-phenyl-glycine	92143	-3.25
12	asn	asparagine	236	-3.38
13	asp	aspartic acid	424	-3.61
14	tyr	tyrosine	1153	-3.68
15	orn	ornithine	389	-3.91
16	trp	tryptophan	1148	-3.91
17	bht	beta-hydroxy-tyrosine	13309269	-3.98
18	abu	2-amino-butyric acid	6657	-4.12
19	glu	glutamic acid	611	-4.27
20	dhpg	3,5-dihydroxy-phenyl-glycine	108001	-4.31
21	hiv	2-hydroxyisovalerate	99823	-4.44
22	dab	2,4-diaminobutyric acid	470	-4.46
23	arg	arginine	232	-4.50
24	lys	lysine	866	-4.62
25	iva	isovaline	94744	-4.81
26	dhb	2,3-dihydroxy-benzoic acid	19	-5.05
27	his	histidine	773	-5.14
28	cys	cysteine	594	-5.16
29	b-ala	beta-alanine	239	-5.21
30	dpr	2,3-diaminopropionic acid	364	-5.23
31	met	methionine	876	-5.34
32	hty	homotyrosine	4153395	-5.53
33	cysA	cysteic acid	25701	-5.56
34	pip	pipecolic acid	849	-5.68
35	hse	homoserine	779	-5.72
36	vol	valinol	79019	-6.07
37	phg	phenyl-glycine	3866	-6.16

Continued on next page

#	Code	Name	PubChem CID	$\log P^A(a^{NRP})$
38	I3CA	indole-3-carboxylic acid	69867	-6.25
39	piz	piperazic acid	2762538	-6.25
40	4ppro	4-propyl-proline	14311265	-6.25
41	aad	2-amino-adipic acid	469	-6.25
42	aeo	2-amino-8-oxo-9,10-decanoate	12917729	-6.25
43	alaol	alaninol	5126	-6.25
44	tcl	(4S)-5,5,5-trichloro-leucine	73811698	-6.25
45	b-lys	beta-lysine	392	-6.25
46	sal	salicylic acid	338	-6.25
47	bmt	4-butenyl-4-methyl threonine	73746095	-6.25
48	cap	capreomycidine	4627981	-6.25
49	kyn	kynurenine	846	-6.25
50	phe-ac	phenylacetate	4409936	-6.25
51	dht	dehydro-threonine	219	-6.25
52	GlyA	glycolic acid	757	-6.25
53	HICA	leucic acid	92779	-6.25
54	lyserg	lysergic acid	622688	-6.25
55	L3Pal	3-(3-pyridyl)-L-alanine	152953	-6.25
56	hyv	4-hydroxy-L-valine	15927951	-6.25
57	LDAP	diaminopimelic acid	865	-6.25
58	OIV	2-oxoisovaleric acid	49	-6.25

Supplementary Table S1: Core amino acids supported by Nerpa along with their PubChem CIDs [6] and scores

$\log P^A_{match}(s)$				
$s = 100$	$s \in [90, 100)$	$s \in [80, 90)$	$s \in [70, 80)$	$s \in (0, 70)$
-0.13	-0.19	-0.81	-1.41	-1.70

$\log P^M_{match}(m^{NRP})$		$\log P^M_{mismatch}(m^{NRP})$		$\log P^M(m^{NRP})$
$m^{NRP} = 1$	$m^{NRP} = -1$	$m^{NRP} = 1$	$m^{NRP} = -1$	$m^{NRP} = 1$
-0.03	-0.02	-3.92	-3.69	-2.15

$\log P^E_{match}(e^{NRP})$		$\log P^E_{mismatch}(e^{NRP})$		$\log P^E(e^{NRP})$
$e^{NRP} = 1$	$e^{NRP} = -1$	$e^{NRP} = 1$	$e^{NRP} = -1$	$e^{NRP} = 1$
-0.33	-0.05	-3.05	-1.26	-1.68

$P_{insertion}$	$P_{deletion}$
-2.69	-3.21

Supplementary Table S2: Nerpa scoring parameters. See Methods for details.

Supplementary Note

Inspection of GARLIC false positive identifications

Due to the score normalization, GARLIC is biased towards small nonribosomal peptides (NRPs) and short biosynthetic gene clusters (BGCs). The normalization step divides the raw score of a given BGC-NRP pair by the maximum of the raw scores of self-aligned BGC-BGC and NRP-NRP pairs [7]. In a search against a large NRP database, amino acid specificities predicted from a BGC with one or two A domains may perfectly match the amino acid sequence of an unrelated short NRP by random chance. While the raw scores of such erroneous BGC-NRP pairs are low, their normalized scores reach the maximal possible value (1.0) and deteriorate GARLIC's false discovery rate.

After searching 194 BGCs from MIBiG$_{\text{NRP}}$ against 8,449 putative NRPs from pNRPdb+, GARLIC linked 27 BGCs to their putative products with the maximal possible score (Section 2.3 in the main text). Most of these BGCs are short; almost all of the links (23 out of 27) are wrong. For example, GARLIC incorrectly linked anabaenopeptin NZ857 BGC (MIBiG BGC0001479) from *Nostoc punctiforme* with the cyclo(D-Tyr-L-Leu) compound (NPA015329) produced by *Bacillus* sp. The BGC contains two A domains; the GARLIC pipeline predicted the domain specificities as Tyr and Leu using PRISM v2 [8] and matched them with the Tyr-Leu sequence decomposed from the compound structure using GRAPE [7]. Despite seeming a perfect match, this BGC-compound pair conceals several inconsistencies. The top three PRISM specificity predictions for the first A domain are: Tyr (with a score of 718.8), Phe (698.5), and Trp (621.6); for the second A domain are: Leu (615.3), Arg (592.4), and Lys (550.4). The small score margin indicates ambiguity, i.e., the very top predictions could be incorrect. In particular, antiSMASH v5 [9] reports the two domains specificities as Phe (with the maximal score 100) and Lys (80). In addition, PRISM and antiSMASH predict a single E domain in the second module of the BGC suggesting the L-configuration of the first amino acid and the D-configuration of the second amino acid that contradicts the compound stereochemistry (D-Tyr; L-Leu). In contrast to Nerpa, GARLIC ignores the certainty of A domain specificity predictions and compounds stereochemistry, leading to erroneously high scores for random matches such as BGC0001479-NPA015329.

References

[1] Kim D Pruitt, Tatiana Tatusova, and Donna R Maglott. NCBI reference sequence (RefSeq): a curated non-redundant sequence database of genomes, transcripts and proteins. *Nucleic acids research*, 33(suppl_1):D501–D504, 2005.

[2] Brian D Ondov, Nicholas H Bergman, and Adam M Phillippy. Interactive metagenomic visualization in a web browser. *BMC bioinformatics*, 12(1):1–10, 2011.

[3] Xiao-Hua Chen, Alexandra Koumoutsi, Romy Scholz, and Rainer Borriss. More than anticipated–production of antibiotics and other secondary metabolites by bacillus amy-

loliquefaciens fzb42. *Journal of molecular microbiology and biotechnology*, 16(1-2):14–24, 2009.

[4] Fatoumata Tambadou, Thibault Caradec, Anne-Laure Gagez, Antoine Bonnet, Valérie Sopéna, Nicolas Bridiau, Valérie Thiéry, Sandrine Didelot, Cyrille Barthélémy, and Romain Chevrot. Characterization of the colistin (polymyxin e1 and e2) biosynthetic gene cluster. *Archives of microbiology*, 197(4):521–532, 2015.

[5] Mohammad R Seyedsayamdost, Matthew F Traxler, Shao-Liang Zheng, Roberto Kolter, and Jon Clardy. Structure and biosynthesis of amychelin, an unusual mixed-ligand siderophore from amycolatopsis sp. aa4. *Journal of the American Chemical Society*, 133(30):11434–11437, 2011.

[6] Sunghwan Kim, Paul A Thiessen, Evan E Bolton, Jie Chen, Gang Fu, Asta Gindulyte, Lianyi Han, Jane He, Siqian He, Benjamin A Shoemaker, et al. Pubchem substance and compound databases. *Nucleic acids research*, 44(D1):D1202–D1213, 2016.

[7] C. A. Dejong, G. M. Chen, H. Li, C. W. Johnston, M. R. Edwards, P. N. Rees, M. A. Skinnider, A. L. Webster, and N. A. Magarvey. Polyketide and nonribosomal peptide retro-biosynthesis and global gene cluster matching. *Nat Chem Biol*, 12(12):1007–1014, Dec 2016.

[8] Michael A Skinnider, Chad W Johnston, Robyn E Edgar, Chris A Dejong, Nishanth J Merwin, Philip N Rees, and Nathan A Magarvey. Genomic charting of ribosomally synthesized natural product chemical space facilitates targeted mining. *Proceedings of the National Academy of Sciences*, 113(42):E6343–E6351, 2016.

[9] Kai Blin, Simon Shaw, Katharina Steinke, Rasmus Villebro, Nadine Ziemert, Sang Yup Lee, Marnix H Medema, and Tilmann Weber. antismash 5.0: updates to the secondary metabolite genome mining pipeline. *Nucleic acids research*, 47(W1):W81–W87, 2019.
